# Host–Guest Interaction Study of Olmesartan Medoxomil with β-Cyclodextrin Derivatives

**DOI:** 10.3390/molecules29102209

**Published:** 2024-05-08

**Authors:** Minodora Andor, Claudia Temereancă, Laura Sbârcea, Adriana Ledeți, Dana Emilia Man, Cristian Mornoș, Amalia Ridichie, Denisa Cîrcioban, Gabriela Vlase, Paul Barvinschi, Angela Caunii, Renata-Maria Văruţ, Cristina Maria Trandafirescu, Valentina Buda, Ionuț Ledeți, Matilda Rădulescu

**Affiliations:** 1 Faculty of Medicine, “Victor Babeş” University of Medicine and Pharmacy, 2 Eftimie Murgu Square, 300041 Timisoara, Romania; andor.minodora@umft.ro (M.A.); man.dana@umft.ro (D.E.M.); cmornos@cardiologie.ro (C.M.); radulescu.matilda@umft.ro (M.R.); 2 Faculty of Industrial Chemistry and Environmental Engineering, University Politehnica Timisoara, 2 Victoriei Square, 300006 Timisoara, Romania; claudia.temereanca@student.upt.ro (C.T.); ionut.ledeti@umft.ro (I.L.); 3 Faculty of Pharmacy, “Victor Babeş” University of Medicine and Pharmacy, 2 Eftimie Murgu Square, 300041 Timisoara, Romania; sbarcea.laura@umft.ro (L.S.); amalia.ridichie@umft.ro (A.R.); circioban.denisa@umft.ro (D.C.); caunii.angelica@umft.ro (A.C.); trandafirescu.cristina@umft.ro (C.M.T.); buda.valentina@umft.ro (V.B.); 4 Advanced Instrumental Screening Center, Faculty of Pharmacy, “Victor Babes” University of Medicine and Pharmacy Timisoara, 2 Eftimie Murgu Square, 300041 Timişoara, Romania; 5 Research Centre for Thermal Analysis in Environmental Problems, West University of Timisoara, Pestalozzi Street 16, 300115 Timisoara, Romania; gabriela.vlase@e-uvt.ro; 6 Faculty of Physics, West University of Timisoara, 4 Vasile Parvan Blvd, 300223 Timisoara, Romania; pc_barvi@yahoo.fr; 7 Faculty of Pharmacy, University of Medicine and Pharmacy Craiova, 2–4 Petru Rares Str., 200349 Craiova, Romania; rennata_maria@yahoo.com

**Keywords:** olmesartan medoxomil, cyclodextrins, inclusion complex, molecular encapsulation, solubility enhancement, spectroscopic methods, thermoanalytical techniques

## Abstract

Olmesartan medoxomil (OLM) is a selective angiotensin II receptor antagonist used in the treatment of hypertension. Its therapeutic potential is limited by its poor water solubility, leading to poor bioavailability. Encapsulation of the drug substance by two methylated cyclodextrins, namely randomly methylated β-cyclodextrin (RM-β-CD) and heptakis(2,3,6-tri-O-methyl)-β-cyclodextrin (TM-β-CD), was carried out to overcome the limitation related to OLM solubility, which, in turn, is expected to result in an improved biopharmaceutical profile. Supramolecular entities were evaluated by means of thermoanalytical techniques (TG—thermogravimetry; DTG—derivative thermogravimetry), spectroscopic methods including powder X-ray diffractometry (PXRD), universal-attenuated total reflectance Fourier-transform infrared (UATR-FTIR) and UV spectroscopy, saturation solubility studies, and by a theoretical approach using molecular modeling. The phase solubility method reveals an *A_L_*-type diagram for both inclusion complexes, indicating a stoichiometry ratio of 1:1. The values of the apparent stability constant indicate the higher stability of the host–guest system OLM/RM-β-CD. The physicochemical properties of the binary systems are different from those of the parent compounds, emphasizing the formation of inclusion complexes between the drug and CDs when the kneading method was used. The molecular encapsulation of OLM in RM-β-CD led to an increase in drug solubility, thus the supramolecular adduct can be the subject of further research to design a new pharmaceutical formulation containing OLM, with improved bioavailability.

## 1. Introduction

In recent decades, cyclodextrins (CDs) have gained increasing attention because of their widespread applications in the pharmaceutical field, biomedicine, food, cosmetic, textile, and chemical industries, agricultural production, and for analytical purposes. CDs are a family of cycling oligosaccharides consisting of D-glucopyranose units connected by α-(1,4)-glycosidic bonds. They present a hollow, truncated cone-shaped structure, with a hydrophilic outer surface, responsible for their water solubility, and a hydrophobic inner cavity that allows the formation of host–guest inclusion complexes (IC) with drug substances of appropriate size [1,2,3,4,5,6]. Native CDs, α, β and γ, containing six, seven and eight glucose units, are frequently used as pharmaceutical excipients, having the GRAS status (Generally Regarded as Safe) from the FDA (Food and Drug Administration) and approval for use also from the EMA (European Medicines Agency) [7,8,9]. The encapsulation of bioactive molecules in the cavity of CD leads to considerable improvements in the physicochemical and biopharmaceutical characteristics of the embedded compounds, CDs acting mainly as solubilizing agents for active substances with poor aqueous solubility [8,10,11,12,13,14,15,16]. Furthermore, CDs offer additional advantages, including improving the physical and chemical stability of guests, their antioxidant and biological activity, reducing irritation at the administration site, masking unpleasant taste or smell, and preventing drug–drug or drug–excipient interaction [17,18,19,20,21,22,23,24,25]. β-cyclodextrin (β-CD) has gained pharmaceutical relevance owing to its economic advantages and internal cavity size suitable for a great number of bioactive molecules, but it exhibits limited water solubility. To overcome this drawback, derivative CDs have been developed, among them methylated β-CDs [22,26,27]. 

**Figure 1:**
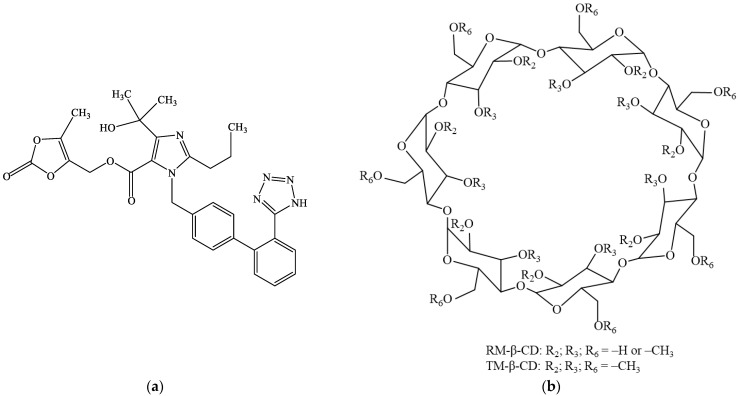
Chemical structure of olmesartan medoxomil (OLM) (**a**); RM-β-CD and TM-β-CD (**b**).

Olmesartan medoxomil (OLM), *(5-methyl-2-oxo-2H-1,3-dioxol-4-yl)methyl 4-(2-hydroxypropan-2-yl)-2-propyl-1-{[2′-(1H-1,2,3,4-tetrazol-5-yl)-[1,1′-biphenyl]-4-yl]methyl}-1H-imidazole-5-carboxylate* (Figure 1a) is an angiotensin II receptor type 1 blocker used for the treatment of hypertension [28,29]. OLM also has antioxidant activity and a renal protective effect that is independent of its antihypertensive effect [28,30]. OLM is a prodrug that is hydrolyzed into its active form, olmesartan, during absorption from the gastrointestinal tract [31,32]. OLM is practically insoluble in water (7.75 mg L^−1^ at 25 °C) and exhibits high lipophilicity (log *P* of 4.31), thus being a BCS (Biopharmaceutics Classification System) Class II candidate; its bioavailability after oral administration is approximately 26% [32,33].

Several approaches have been explored to improve the dissolution rate and bioavailability of OLM, including the formulation of the self-microemulsifying drug delivery system (SMEDDS) with Acrysol EL135 as oil, Tween 80 as surfactant and Transcutol P as co-surfactant [31], ternary solid dispersion of the drug with hydroxypropyl-β-cyclodextrin (HP-β-CD) and N-methyl-D-glucamine (MG) prepared by mechanochemistry [33], nano-sized formulation as lipid-based nanoparticles and nanosuspension [29] and sustained-release tablets using composites of anionic sulfobutyl ether-β-cyclodextrin (SBE-β-CD) and cationic spray dried chitosan (SD-CS) [28]. 

Complexation of active pharmaceutical ingredients with CD is one of the valuable strategies used to enhance the solubility of poorly water-soluble drugs, the dissolution rate, and consequently their bioavailability [28,34]. Thakkar et al. [35] have investigated the inclusion of OLM in HP-β-CD cavity and reported significantly higher saturation solubility, dissolution rate, and intestinal permeation for OLM/HP-β-CD binary systems.

The aim of this study was to investigate the inclusion complexation of OLM with two methylated β-CD, randomly methylated β-cyclodextrin (RM-β-CD) and heptakis(2,3,6-tri-O-methyl)-β-cyclodextrin (TM-β-CD) (Figure 1b) for improving drug solubility. UV spectroscopy, thermogravimetry (TG), derivative thermogravimetry (DTG), powder X-ray diffractometry (PXRD), UATR-FTIR spectroscopy were used to characterize the supramolecular adducts both in solution and in solid state. Molecular modeling was also performed in order to evaluate the inclusion complexes geometry. Saturation solubility studies were further carried out to assess the physicochemical performance of the binary systems.

## 2. Results and Discussion

### 2.1. Phase Solubility Studies

**Figure 2:**
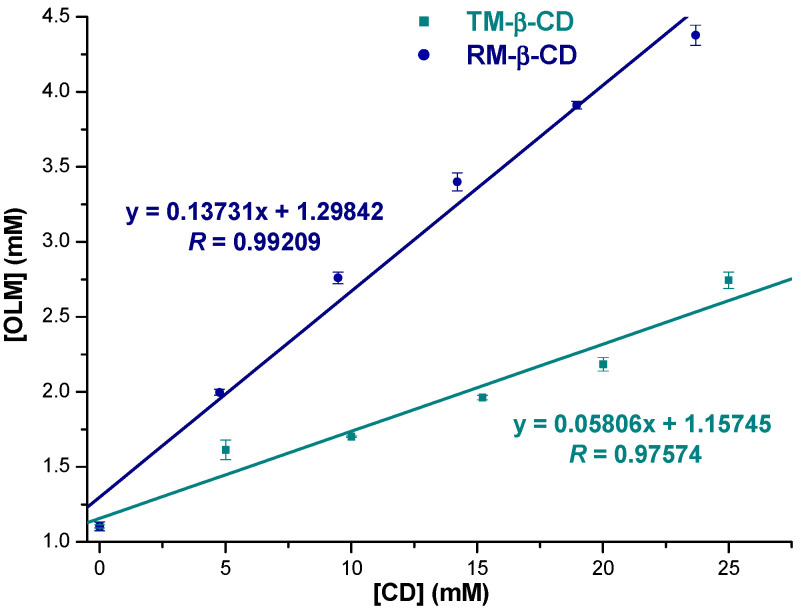
Phase solubility diagram of OLM with RM-β-CD (blue circle) and TM-β-CD (dark cyan squares) in 0.1 M phosphate buffer, pH 7.4, at 25 °C.

Phase solubility studies were conducted to estimate the molar ratio and stability constant for the OLM inclusion complexes with RM-β-CD and TM-β-CD. Phase solubility diagrams at 25 °C, in 0.1 M phosphate buffer (pH 7.4) are presented in Figure 2. The apparent solubility of OLM increased linearly with increasing concentration of both RM-β-CD (R = 0.99209) and TM-β-CD (R = 0.97574) in the 0–25 mM concentration range, confirming the interaction between the drug substance and CDs. The linear relationship indicates *A_L_*-type phase solubility diagrams according to the Higuchi and Connors classification [36] for both host molecules, suggesting the formation of soluble IC in a 1:1 molar ratio between the drug and the CDs under experimental conditions. Additionally, the slope values of the phase solubility diagrams were less than 1.0 (0,13731 for RM-β-CD; 0,05806 for TM-β-CD). The apparent stability constant *(K*_1*:*1_) of the inclusion complexes was calculated from the slope of the phase solubility diagram, using Equation (1) and the results emphasized that OLM strongly interacts with RM-β-CD (144.24 ± 3.85 M^−1^) that TM-β-CD (55.86 ± 1.74 M^−1^). Furthermore, data also suggested that RM-β-CD possesses a higher solubilization capacity than TM-β-CD for the guest molecule in 0.1 M phosphate buffer (pH 7.4). 

### 2.2. Job’s Method

**Figure 3:**
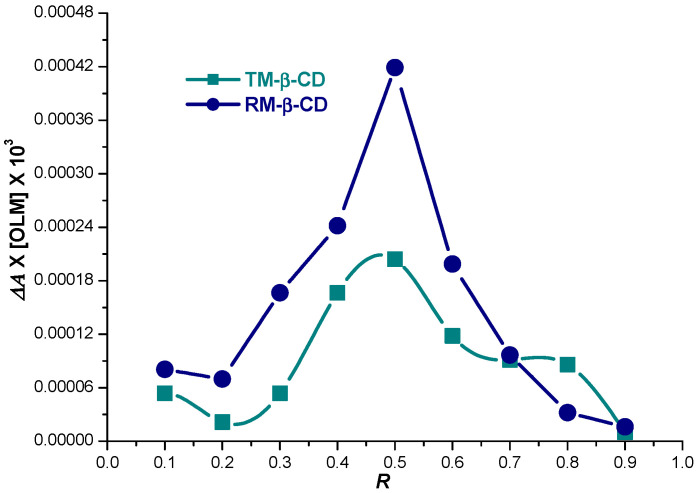
Job’s plot for OLM/RM-β-CD (blue circle) and OLM/TM-β-CD (dark cyan squares) inclusion complexes in 0.1 M phosphate buffer, pH 7.4.

The stoichiometry of the ICs of OLM with methylated CDs was also evaluated by means of Job’s method in order to confirm the results of phase solubility studies. Job’s plot for OLM and each CD was obtained by plotting *ΔA*∙[OLM] vs. R, where *ΔA* (*ΔA = A − A*_0_) represents the differences in OLM absorbance in the presence (*A*) and the absence (*A*_0_*)* of CDs, and R stands for OLM mole fraction, (R = [OLM]/[OLM] + {CD]). According to Job’s method, the IC stoichiometry is provided by the value of the mole fraction R that correlates with the maximum concentration of the IC [37]. The maximum difference in OLM absorbance is noticed for a OLM mole fraction of 0.5 in the presence of both RM-β-CD and TM-β-CD as Figure 3 reveals, suggesting the main stoichiometry is 1:1 for both CDs. These findings are in agreement with the results obtained from phase solubility studies.

### 2.3. Encapsulation Efficiency and Loading Efficiency Analysis 

Encapsulation efficiency (EE) is expressed as the mass percentage of the incorporated OLM to the total amount of drug added in the inclusion complex, while the loading efficiency (LE) is defined as the mass ratio of encapsulated OLM to the mass of IC [38,39]. The OLM content in OLM/CD ICs was assessed spectrophotometrically using the calibration curve. The EE and LE values for OLM/RM-β-CD KP and OLM/TM-β-CD KP were found to be 82.46 ± 2.16%, 24.60 ± 1.34% and 76.77 ± 1.35%, 22.97 ± 1.18%. These results are in agreement with those obtained from phase solubility studies emphasizing a better interaction between the drug substance and RM-β-CD.

### 2.4. Molecular Modeling Studies

The molecular docking experiments predicted the complexation process through the noncovalent interactions between OLM and host molecules in a 1:1 molar ratio. The molecular docking studies were conducted using Autodock 4.2.6 software together with AutoDockTools [40].

AutoDock is a suite of automated docking tools designed to predict how small molecules, such as substrates or drug candidates, bind to a receptor of known 3D structure. According to AutoDock, the binding energy is the sum of the intermolecular forces that act on the receptor-ligand complex.

Binding energy=A+B+C−D

where A denotes the final intermolecular energy + van der Walls energy (vdW) + hydrogen bonds + desolvation energy + electrostatic energy (kcal mol^−1^), B denotes the final total internal energy (kcal mol^−1^), C denotes the torsional free energy (kcal mol^−1^), D denotes the energy of the unbound system (kcal mol^−1^).

The binding free energy values were calculated as −6.53 kcal mol^−1^ for the OLM/RM-β-CD adduct, and −7.92 kcal mol^−1^ for the OLM/TM-β-CD system.

After Autodock 4.2.6 redocking, root-mean-square deviation (RMSD) values were calculated, obtaining low RMSD values (all of them are ≤ 0.25 Å) that suggest that our docking methodology is robust.

**Figure 4:**
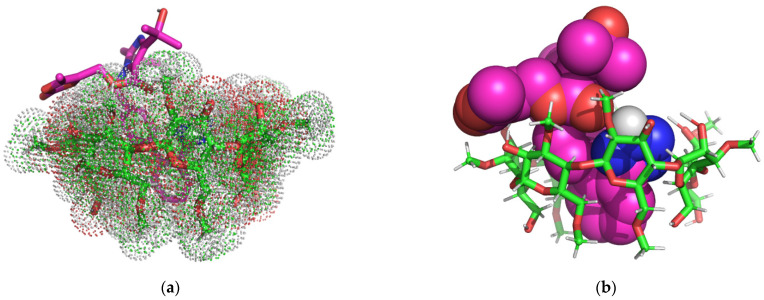

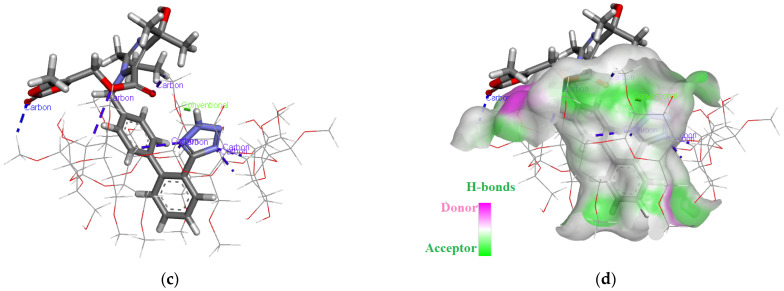
OLM/RM-β-CD inclusion complex simulation (1:1 molar ratio). Images (**a**,**b**) present the binary system from the secondary face of RM-β-CD cavity. The OLM molecule is rendered in sticks colored by element (**a**), spheres colored by element (**b**), while RM-β-CD is represented in red/green/white dots (**a**), in sticks (**b**); image (**c**) shows polar/hydrophobic contacts between guest, represented in sticks colored by element, and host, represented in lines; image (**d**) shows the H-bond surface interaction OLM/RM-β-CD.

In Figure 4 is represented the theoretical OLM/RM-β-CD IC as rendered in the PyMOL molecular visualization system [41], simulated in a 1:1 molar ratio. 

The 3D Images of the OLM/RM-β-CD (1:1) interaction revealed the presence of seven non-classical carbon-hydrogen bonds (2.07 Å–2.89 Å) involving the 2-oxo-1,3-dioxol heterocycle, the oxygen atoms of the carboxylate group, the 1,4 nitrogen atoms of the tetrazole heterocycle from OLM structure and the C-H of RM-β-CD glucopyranose residue. The 2H hydrogen of the tetrazole heterocycle forms a classical hydrogen bond with a distance of 1.96 Å with the second position oxygen of a carbohydrate moiety.

**Figure 5:**
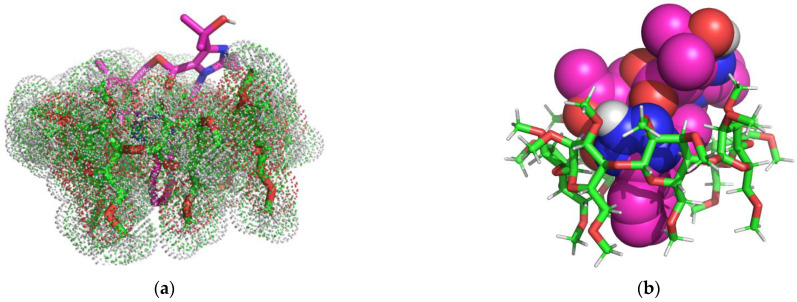

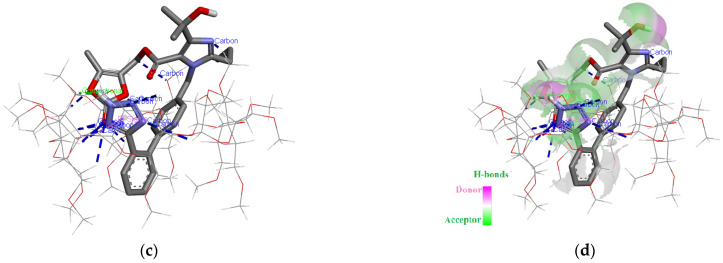
OLM/TM-β-CD inclusion complex simulation for 1:1 molar ratio. Images (**a**,**b**) show the binary system from the secondary face of TM-β-CD cavity. OLM guest molecule is represented in sticks colored by element, while TM-β-CD, in red/green/white dots (**a**); OLM is presented in spheres colored by element and TM-β-CD, in sticks red/green/white colored (**b**). Image (**c**) shows polar/hydrophobic contacts between OLM, represented in sticks colored by element, and TM-β-CD represented in lines. Image (**d**) shows H-bond surface interaction between guest and host molecules.

Figure 5 shows 1:1 OLM/TM-β-CD binary system, as rendered in the PyMOL molecular visualization system [41]. 

Analyzing the 3D images of the OLM/TM-β-CD complex in a molar ratio of 1:1, the formation of fifteen non-classical carbon-hydrogen bonds (1.92 Å–2.83 Å), host–guest interactions, involving the 2-oxo-1,3-dioxol heterocycle, the first position nitrogen atom of the tetrazole heterocycle and the first nitrogen atom of imidazole heterocycle from OLM structure and the C-H of TM-β-CD glucopyranose residue can be observed. Also, a hydrophobic Pi-sigma electrostatic interaction (2.37 Å) involving the OLM tetrazole heterocycle and TM-β-CD hydrogen atom from the fourth position of a carbohydrate moiety can be observed. The 2H hydrogen of the tetrazole heterocycle forms a classical hydrogen bond with a distance of 2.26 Å with the oxygen from the sixth position of a carbohydrate moiety.

According to the molecular docking studies, the OLM/TM-β-CD complex it was expected to be more stable as its free energy value is smaller than that of the OLM/RM-β-CD complex and also has a greater number of intermolecular bonds. On the contrary, the phase solubility studies carried out in phosphate buffer, pH 7.4 and the encapsulation efficiency and loading efficiency analyses bring out a more intense interaction between OLM and RM-β-CD given the values of the stability constants, EE and LE.

### 2.5. FTIR Spectroscopy

**Figure 6:**
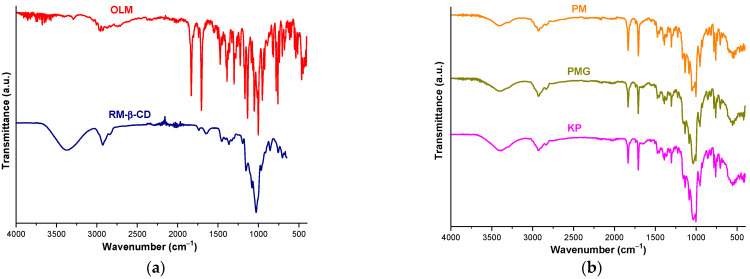

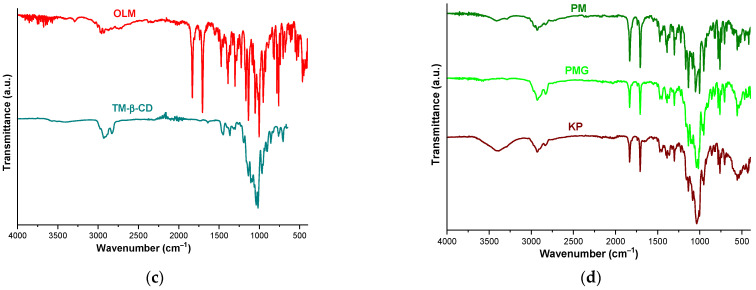
UATR-FTIR spectra of OLM, RM-β-CD (**a**) and their PM, PMG and KP products (**b**); OLM, TM-β-CD (**c**), and their PM, PMG and KP compounds (**d**).

The UATR-FTIR spectra of the OLM, CDs, OLM/RM-β-CD and OLM/TM-β-CD binary systems obtained by mixing the substances with a pestle (PMG), with a spatula (PM) and using the kneading method (kneaded products, KP) are presented in Figure 6 and were evaluated to assess the interaction of the drug substance with CDs. 

The UATR-FTIR spectrum of OLM reveals prominent characteristic absorption bands at 1830 cm^−1^ assigned to the carbonyl stretching vibration of lactone and at 1705 cm^−1^ corresponding to the C=O stretching vibration from the ester group. Other characteristic bands of OLM are noticed at 1225 and 1167 cm^−1^ due to C-O stretching from lactone and ester groups, at 1551, 1503 and 1474 cm^−1^ assigned to C=C stretching vibration of the aromatic ring, at 1389 cm^−1^ due to C=N stretching vibration, at 1136 cm^−1^ attributed to C-O stretching vibration from tertiary alcohol, at 953, 826, 816, 781, 770 cm^−1^ corresponding to C_ar_-H bending. The C-N stretching vibration appears at 1302 cm^−1^, the NNN bending vibration arises at 1002 cm^−1^ and the NH wagging is shown at 760 and 741 cm^−1^ [42,43]. 

The spectral pattern of RM-β-CD exhibits a broad absorption band in the spectral region of 3600–3100 cm^−1^ that corresponds to the O-H stretching vibration of the nonmethylated hydroxyl groups and a large region below 1500 cm^−1^ exhibiting distinct peaks characteristic of the CD ring [12,19,44].

FTIR spectrum of OLM/RM-β-CD PM reveals the presence of OLM characteristic bands assigned to C=C stretching vibration of the aromatic ring (1551, 1503, 1474 cm^−1^), C-N and C=N stretching vibrations (1302, 1389 cm^−1^), C-O stretching from lactone (1225 cm^−1^), NH wagging (760, 741 cm^−1^) and C_ar_-H bending from 953 and 826 cm^−1^. Some differences in terms of bands position are noticed in the PM FTIR spectrum compared to that of pure OLM. Thus, OLM bands assigned to the carbonyl stretching vibration of lactone and ester groups are shifted from 1830 and 1705 cm^−1^ in pure OLM to 1832 and 1707 cm^−1^ in PM, the OLM band of ester C-O stretching is displaced at 1165 cm^−1^, the band attributed to C-O stretching vibration from tertiary alcohol is noticed at 1134 cm^−1^ and the one of NNN bending vibration is observed at 1003 cm^−1^. Also, the OLM characteristic band from 1090 cm^−1^ is displaced at 1086 cm^−1^ and those of C_ar_-H bending from 816, 781 and 770 cm^−1^ in pure OLM are shifted to 818, 783 and 768 cm^−1^ in PM spectrum. 

Important differences are noticed in the FTIR spectra of binary systems PMG and KP compared with those of the parent compound in terms of intensity and position of the spectral bands. Specifically, the characteristic OLM bands of ester and lactone carbonyl stretching are displaced at 1832 and 1707 cm^−1^ in both PMG and KP and the bands assigned to CO stretching shifted from 1225 and 1167 cm^−1^ in pure OLM to 1227 and 1155 cm^−1^ in both PMG and KP. Also, the characteristic band of OLM from 1090 cm^−1^ is observed at 1084 cm^−1^ in the PMG and KP spectra and the bands attributed to the aromatic bending of C-H shifted from 816 and 781 cm^−1^ in pure OLM to 818 and 783 cm^−1^ in the KP spectrum. In addition, the OLM characteristic bands from 1281, 1069 and 1016 cm^−1^ disappeared in both PMG and KP spectra. 

The FTIR TM-β-CD characteristic peaks appear at 2929 cm^−1^ (symmetric and asymmetric C-H stretching vibration from CH_2_), at 1365 cm^−1^ (C-H bending from CH_2_), at 1016 cm^−1^ (C-C-O stretching vibration). In addition, characteristic bands attributed to C–O–C stretching vibrations are shown in the spectral region of 1076-1022 cm^−1^ [12,45].

The FTIR profile of OLM/TM-β-CD PM shows OLM characteristic bands corresponding to C=O stretching vibration from lactone (1830 cm^−1^), C=N and C-N stretching vibrations (1389, 1302 cm^−1^), C_ar_-H bending from 953 and 826 cm^−1^, NH wagging (760, 741 cm^−1^) and C=C stretching vibration of the aromatic ring (1551, 1503, 1474 cm^−1^). OLM characteristic bands at 1258 and 1090 cm^−1^ are also noticed in PM spectrum. The OLM band assigned to stretching of ester C=O from 1705 cm^−1^ in pure OLM is shifted to 1707 cm^−1^ and those corresponding to C-O stretching from lactone and ester are displaced at 1227 and 1165 cm^−1^. Also, the C-O stretching vibration of tertiary alcohol produced intense band at 1134 cm^−1^ in PM, the band of NNN bending vibration is observed at 1003 cm^−1^ and the bands from 1069, 816, 781 and 770 cm^−1^ in pure OLM are shifted to 1067, 818, 783 and 768 cm^−1^ in PM.

Differences are also observed in the FTIR spectra of binary products PMG and KP when compared with the spectra of the parent compounds. Thus, the characteristic OLM bands that correspond to the stretching of C=O from the lactone and ester groups are displaced at 1832 and 1707 cm^−1^ in both PMG and KP and decrease their intensity. The band assigned to the stretching of lactone CO from 1225 cm^−1^ in pure OLM shifted to 1227 cm^−1^ in PMG and the stretching of ester CO at 1167 cm^−1^ in OLM is displaced at 1163 cm^−1^ in PMG and 1157 cm^−1^ in the KP and is markedly reduced in intensity. Also, the OLM characteristic band from 1090 cm^−1^ shifted to 1088 cm^−1^ in PMG and to 1084 cm^−1^ in KP and the OLM band from 1069 cm^−1^ is noticed at 1065 in the PMG spectrum and disappeared in the KP spectral pattern. Additionally, the peak from 1258 cm^−1^ in the OLM spectral profile is no longer present in either PMG or KP spectra, while a new broad band is noticed at 3395 cm^−1^ in the KP spectrum without a corresponding band in the IR spectra of pure substances, probably attributed to the H intermolecularly bonded O-H stretching.

The findings of FTIR studies highlight the decrease in intensity of OLM characteristic bands together with a small displacement to other wavenumbers of some OLM peaks in the binary systems obtained by gently mixing with the spatula of the parent substances. Additionally, in both PMG and KP spectra the disappearance of several bands along with a greater shift to different wavenumbers of OLM bands compared to PM is observed. These data emphasize the interaction between OLM and both RM-β-CD and TM-β-CD in the binary products obtained by mixing using the pestle and employing the kneading method and also suggest a potential interaction in OLM/CDs PM.

### 2.6. Thermal Analysis

**Figure 7:**
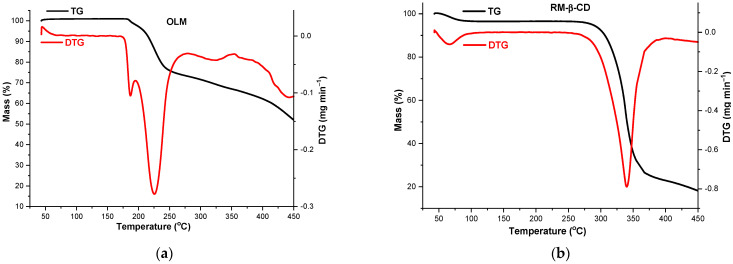

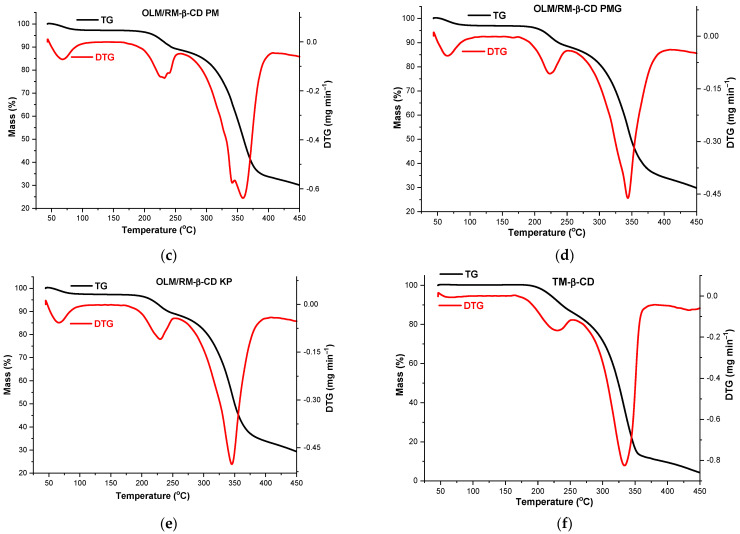

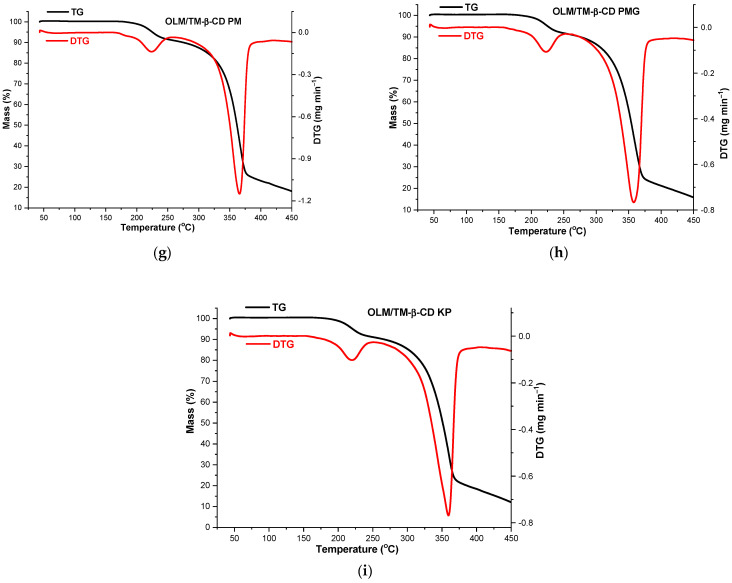
TG/DTG thermal curves of OLM (**a**); RM-β-CD (**b**); OLM/RM-β-CD PM (**c**); OLM/RM-β-CD PMG (**d**); OLM/RM-β-CD KP (**e**); TM-β-CD (**f**); OLM/TM-β-CD PM (**g**); OLM/TM-β-CD PMG (**h**) and OLM/TM-β-CD KP (**i**) under nitrogen flow of 100 mL min^−1^, at a heating rate of 10 °C min^−1^.

Thermal analysis was also used to assess the interaction between OLM and CDs in the solid state. The thermal behavior of OLM, CDs, OLM/RM-β-CD and OLM/ TM-β-CD PM, PMG and KP was investigated in an inert atmosphere using TG and DTG, and the thermal curves of the compounds are shown in Figure 7.

The thermoanalytical profile of OLM reveals a good thermal stability of the drug substance with no mass loss observed up to 174 °C (Figure 7a), temperature value that indicates the onset of OLM decomposition. Then, a continuous process of mass loss is observed up to 450 °C (*Δm* = 48.1%), as a result of thermal-induced degradation. The DTG curve shows three distinct regions, indicating that OLM decomposition occurs in three steps. The first process takes place in the temperature range 174–195 °C (DTG_max_ = 187 °C, *Δm* = 1.9%), the second is observed between 195 and 278 °C (DTG_max_ = 226 °C, *Δm* = 24.9%) and the third is noticed in the temperature range 283–353 °C (DTG_max_ = 323 °C, *Δm* = 6.5%). Above this temperature value OLM decomposition continues until a residual mass of 51.9% at 450 °C.

The thermoanalytical profile of RM-β-CD indicates a small mass loss between 40 °C and 105 °C (DTG_max_ = 67 °C, *Δm* = 5.2%) corresponding to the CD crystallization water loss, specifically, 3.9 water molecules (Figure 7b). The dehydration process is followed by a stability stage in the temperature range 105–260 °C; between 260 and 450 °C the mass loss continues and the degradation process occurs (DTG_max_ = 340 °C, *Δm* = 76.7%) [12,19]. 

Important differences are observed in the thermal profile of OLM/RM-β-CD binary systems PM, PMG and KP compared to those of the pure compounds. OLM/RM-β-CD PM and PMG thermal profiles (Figure 7c,d) indicate a mass loss of 2.8% between 40 and 120 °C (DTG_max_ = 68 °C for PM, DTG_max_ = 65 °C for PMG), attributed to CD dehydration process with the loss of 2.9 water molecules followed by a stability stage between 120 and 170 °C. In the temperature range of 170–410 °C, the decomposition occurs in two stages, the first between 170 and 255 °C (DTG_max_ = 232 °C, *Δm* = 8.2%) and the second between 255 and 410 °C (DTG_max_ = 359 °C, *Δm* = 55.9%) in the case of PM. OLM/RM-β-CD PMG thermoanalytical curves also exhibit the decomposition in two steps above 170 °C, the first in the temperature range 170–250 °C (DTG_max_ = 224 °C, *Δm* = 8.4%) and the second between 250 and 405 °C (DTG_max_ = 344 °C, *Δm* = 54.8%). In both PM and PMG, the mass loss process continues until a residual mass of 30% at 450 °C. OLM/RM-β-CD KP thermal curves indicate a small mass loss between 40 and 110 °C (DTG_max_ = 66 °C, *Δm* = 2.5%) due to the loss of water from CD, totaling 2.6 water molecules. In the temperature range 110–175 °C, the stability of the molecular adduct is observed, followed by continuous mass loss in two stages, the first one between 175 and 252 °C (DTG_max_ = 230 °C, *Δm* = 8.2%) and the last in the temperature range 252–405 °C (DTG_max_ = 345 °C, *Δm* = 55.6%) (Figure 7e).

As the TG/DTG curves show, TM-β-CD decomposition starts at approximately 180 °C, indicating a considerably high thermal stability and it takes place in two steps, the first one between 180 and 255 °C (DTG_max_ = 230 °C, *Δm* = 14.2%) and the second one in the temperature range 255–375 °C (DTG_max_ = 334 °C). Above this temperature, the mass loss continues until a residual mass of 4.3% at 450 °C (Figure 7f). 

The thermal profile of OLM/TM-β-CD PM, PMG and KP also exhibits differences compared to that of the parent substances. TG/DTG curves of PM and PMG indicate a thermal stability of the binary systems up to 176 °C and 170 °C, respectively, temperatures at which the decomposition with continuous mass loss starts (DTG peaks at 224 °C and 366 °C for PM; DTG_max_ at 223 and 358 °C for PMG), the mass residues at 450 °C being 18.04% and 15.83% (Figure 7g,h). The thermal curves of KP show a stability stage of the supramolecular adduct up to 175 °C, followed by decomposition in two steps: the first with a small mass loss between 175 and 250 °C (DTG_max_ = 220 °C, *Δm* = 9.2%) and the second in the temperature range 250–400 °C (DTG_max_ = 359 °C, *Δm* = 72.7%). The mass residue of 12.02% at 450 °C is noticed (Figure 7i).

The different thermal behavior of the OLM/CDs KP from that of the pure substances highlighted by the thermoanalytical methods suggests an interaction between OLM and the CDs and the formation of host–guest inclusion complexes, thus supporting the outcomes of the FTIR spectroscopy. Interactions can also be observed for the physical mixtures, even in the case of physical mixtures prepared without grinding, but only gentle mixing with a spatula, but they are weaker than those in the kneaded products. The interaction between OLM and 2 hydroxypropyl-β-CD even in the physical mixtures was also reported by Thakkar et al. [35]. The smaller mass loss in the temperature range of 40–110 °C observed in the case of KP compared to PM and PMG proves a more intense interaction of OLM and RM-β-CD when the kneading method was used, since the driving force for complexation involves the replacement of high-energy water molecule in the host cavity by guest molecules [46].

The thermal profile of the drug substance, CDs and their binary systems was also evaluated in air atmosphere and bring out insights into the guest-host interaction. As TG curve of OLM shows (Figure S1), a small mass increase (*Δm* = 1%) is observed between 40 and 177 °C, most probably as a result of the 2-oxo 1,3-dioxol heterocycle cleavage due to the OLM oxidative degradation. Investigation of the thermal behavior of OLM/RM-β-CD binary systems reveals a mass increase of 0.13% and 0.05% for PMG and KP, respectively, in the temperature range 100–177 °C attributed to the OLM oxidation. Thermoanalytical profiles of OLM/TM-β-CD also indicate a mass increase between 30 and 177 °C in both PMG and KP products, greater in the PMG (Figure S1). These results suggest that 2-oxo 1,3-dioxol heterocycle is situated outside the CDs cavities being exposed to oxidative action an air and sustain the findings of the molecular docking studies. 

### 2.7. Powder X-ray Diffraction

**Figure 8:**
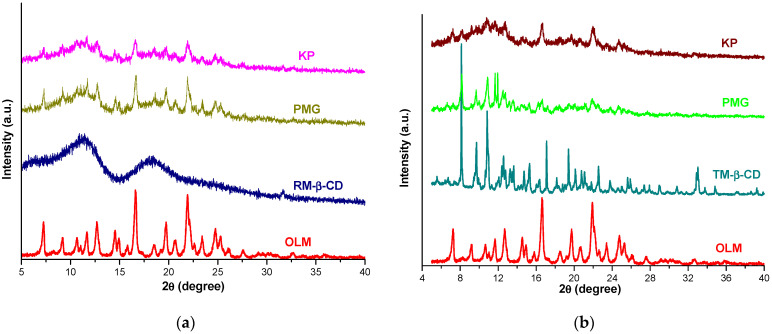
Diffraction pattern of OLM, RM-β-CD, OLM/RM-β-CD PMG and KP (**a**); OLM, TM-β-CD and their binary systems PMG and KP (**b**).

In Figure 8 are shown PXRD patterns of OLM, CDs, OLM/CDs KP and PMG.

The OLM PXRD spectrum presents characteristic diffraction peaks at 7.25; 9.17; 10.64; 11.69; 12.68; 14.54; 16.61; 19.73; 21.92; 24.77 and 25.25 2*θ*, highlighting the crystalline nature of the drug. The diffractometric profile of RM-β-CD shows two broad peaks without characteristic sharp peaks, confirming its amorphous nature [20,47]. The diffraction pattern of OLM/RM-β-CD PMG reveals an important diminution of the characteristic peaks of OLM. An even higher reduction in the intensity of OLM peaks is observed in the PXRD pattern of OLM/RM-β-CD KP along with the disappearance of the characteristic reflections of OLM at 9.17; 10.64 2*θ*, emphasizing an OLM amorphization process, which provides evidence for an interaction between OLM and RM-β-CD [47].

The crystalline nature of TM-β-CD (Figure 8b) is confirmed by the presence of sharp characteristic reflections at 8.14; 9.73; 10.84; 12.58; 15.31; 17.09; 19.40; 22.57 2𝜃 [12,37] in its diffractometric profile. The diffraction pattern of the PMG binary product shows the disappearance of the characteristic peaks of OLM at 12.68; 19.73 and 25.25 2*θ* together with a decrease in both the OLM and TM-β-CD crystalline reflections. OLM/TM-β-CD KP diffractometric profile indicates a greater diminution of OLM and CD characteristic peaks than that of PMG. Furthermore, several characteristic reflections of CD (at 15.31; 17.09; 19.40 2*θ*) and the drug substance (at 12.68; 24.77; 25.25 2*θ*) were absent in the PXRD spectrum of KP supramolecular adduct. 

These results indicate that binary products, PMG and KP, are different in relation with the parent compounds, suggesting the interaction between OLM and CDs resulting in the formation of ICs in the solid state with a simultaneous reduction in OLM crystallinity. 

### 2.8. Solubility Profile of OLM/CD Binary Products

**Figure 9:**
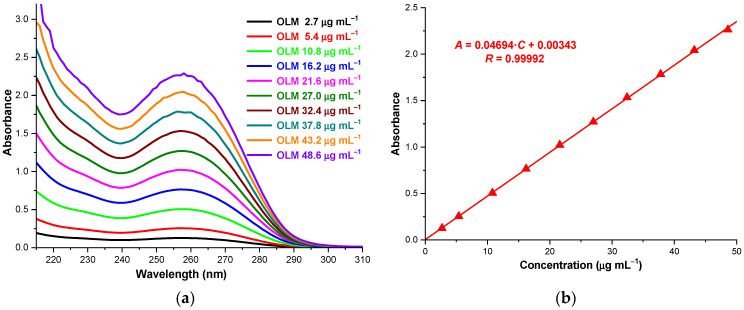
Absorption spectra of OLM solutions in 0.1 M phosphate buffer (pH 7.4), in the spectral range 210–310 (**a**); OLM calibration curve at 257 nm (**b**).

The solubility of the drug substance in host–guest binary systems was assessed by the shake-flask method [19,37,48]. UV spectrophotometry and the calibration curve at 257 nm (Figure 9) was used to evaluate OLM concentration in the saturated solution, as CDs do not show absorption in the spectral range of 210–310 nm. 

The solubility of OLM in the host–guest systems obtained using the kneaded method, calculated as a mean value of five experimental determinations, is 1099.541 ± 0.061 µg mL^−1^ for OLM/RM-β-CD and 659,129 ± 0.024 µg mL^−1^ for OLM/TM-β-CD. The saturation solubility studies proved that RM-β-CD has a greater ability to solubilize OLM than TM-β-CD as the solubility of OLM increased by 1.78-fold in OLM/RM-β-CD KP and by 1.07-fold in OLM/TM-β-CD KP compared to pure OLM (616.404 ± 0.015 µg mL^−1^). 

The OLM solubility was also assessed for the PMG products. The results obtained as a mean value of five experimental determinations were 961.590 ± 0.031 µg mL^−1^ for OLM/RM-β-CD PMG and 628.732 ± 0.028 µg mL^−1^ for OLM/TM-β-CD PMG. The solubility of OLM increased by 1.56-fold in PMG with RM-β-CD and by 1.02-fold in PMG with TM-β-CD. These data suggest the existence of an interaction between the drug substance and the CDs also in the PMG products, weaker than that in KP, thus supporting the results of thermal and spectroscopic techniques. 

Furthermore, the results of the saturation solubility analysis emphasized a better guest-host interaction between OLM and RM-β-CD, sustaining the findings of the phase solubility studies, EE and LE analyses.

## 3. Materials and Methods

### 3.1. Materials

Olmesartan medoxomil (as Pharmaceutical Secondary Standard) (PHR1851) was purchased from Sigma-Aldrich (St. Louis, MO, USA) and CDs, randomly methylated β-cyclodextrin (DS~12) (CY-2004.1) and heptakis(2,3,6-tri-O-methyl)-β-cyclodextrin (CY-2003) were acquired from Cyclolab R&L Ltd. (Budapest, Hungary). The substances were used as received. All the chemicals and reagents used in this study were of analytical grade.

### 3.2. Phase Solubility Studies

Phase solubility analysis was carried out employing the method reported by Higuchi and Connors [36]. Specifically, excessive amounts of OLM were added to different concentrations (0–25 mM) of RM-β-CD and TM-β-CD solutions in 0.1 M phosphate buffer of pH 7.4. and the suspensions obtained were shaken at 25 °C for 5 days to reach equilibrium. The samples were then filtered using a 0.45 µm nylon disk filter and diluted appropriately. The drug concentration was assessed spectrophotometrically at 257 nm, using a Jena Analytik Specord 250 Plus double beam spectrophotometer (Jena, Germany) with matched quartz cells of 1 cm.

The apparent stability constant (*K*_1:1_) of the host–guest systems was obtained from the phase solubility diagram, using the equation:

(1) $$K_{1:1}=\frac{Slope}{S_{0}\left(1-Slope\right)}$$

where *S*_0_ is the solubility of OLM in 0.1 M phosphate buffer (pH 7.4) in the absence of CDs.

### 3.3. Job’s Method

Job’s method was used to assess the stoichiometry of the ICs between OLM and CDs [12,37]. To this end, equimolar 5.37 × 10^−5^ M solutions of OLM and each CD were prepared in 0.1 M phosphate buffer of pH 7.4. A set of solutions in the range of OLM molar ratio 0.0–1.0 was prepared by mixing OLM and each of the two CD solutions in an appropriate proportion. A dilution set of the OLM stock solution was also prepared similarly, using the same solvent. After stirring the solutions, their absorbance was recorded at 257 nm.

### 3.4. Encapsulation Efficiency and Loading Efficiency Analysis

Appropriate amounts of OLM/CD ICs (0.0084 g OLM/RM-β-CD IC and 0.0089 g OLM/TM-β-CD IC) were accurately weighed and mixed with 5 mL of phosphate buffer 0.1 M (pH 7.4) in a 10 mL volumetric flask. After the solvent was added until reaching the mark the solutions were subjected to sonication treatment for 10 min. The solutions were then filtered using 0.45 µm nylon disk filter and appropriately diluted in order to quantify the drug substance using UV spectrophotometric measurements at 257 nm. 

The EE and LE was calculated using the Equations (2) and (3) [38,49]:

(2) $$EE\%=\frac{W_{E}}{W_{T}}\cdot 100$$

(3) $$LE\%=\frac{W_{E}}{W_{I}}\cdot 100$$

where *W_E_* is the amount of encapsulated OLM, *W_T_* is the total amount of OLM added initially during the preparation of each IC and *W_I_* is the mass of each IC.

### 3.5. Molecular Modeling Studies

The three-dimensional coordinates of OLM were downloaded from PubChem (CID 130881) and the geometry was optimized at the theoretical level DFT/B3LYP/6-311G.

To gain insight into the interaction of OLM with RM-β-CD and TM-β-CD, the molecular docking technique was employed. The RM-β-CD structure used in this study was generated from the curated coordinates of the ligand 2QKH (X-ray diffraction, resolution 1.9 A) downloaded from the Protein Data Bank database [50]. Methyl groups were manually added to free hydroxyl groups to obtain a degree of substitution equal to 12 (GaussView 5, Semichem Inc., Shawnee Mission, KS, USA). Substituents were added on the β-cyclodextrin natural core, namely 4 CH_3_ groups on the O-2 position for the 2, 3, 4 and 6 glucose residues, 5 groups on the O-3 for the 1, 2, 4, 5, 7 glucopyranose units and 3 CH_3_ on the O-6 position for the 1, 5, 7 glucopyranose units. For TM-β-CD, the same initial β-cyclodextrin was used, the methyl groups were manually added to all the free OH groups to obtain a fully permethylated structure (GaussView 5, Semichem Inc). CDs were optimized in the same way as OLM (DFT/B3LYP/6-311G).

The stoichiometry of the OLM inclusion complexes was determined using phase solubility studies and Job’s method as a 1:1 OLM:CD molar ratio. 

In the docking protocol, OLM was used as a ligand and CDs as receptors to obtain the best configuration of the complex and to compute the total energy of affinity (kcal mol^−1^). With the ligands and proteins in “.pdbqt”, respectively, pdb format, molecular docking computation was performed in triplicate using Autodock 4.2.6 software together with the AutoDockTools [40]. All calculations were performed in vacuum. For the docking process the Lamarckian genetic algorithm with a population size of 150 and a number of 30 runs were chosen. All other parameters were used with the default values. Molecular modelling figures were generated using PyMol (http://www.pymol.org) [41].

### 3.6. Preparation of the Solid Binary Systems

The OLM inclusion complexes with RM-β-CD and TM-β-CD were obtained using the kneading method and 1:1 OLM:CD molar ratio. For the preparation of OLM/RM-β-CD KP, amounts of 0.1496 g OLM and 0.3527 g RM-β-CD were weighed and the mixture was pulverized in an agate mortar and triturated with 0.5 g ethanol:distilled water (1:1, *m*/*m*). To obtain OLM/TM-β-CD inclusion complex, 0.1414 g of OLM and 0.3618 g TM-β-CD were accurately weighed and the resulting mixture was pulverized and triturated with 0.50 g of ethanol: distilled water (1:1, *m*/*m*) until a homogeneous paste was obtained. The paste was kneaded for 45 min, adding an appropriate amount of solvent during the kneading process to maintain the consistency of the paste. The products obtained were dried at room temperature and then in an oven at 40 ° C for 24 h. Finally, the kneaded products were pulverized and passed through a 75 µm size sieve. The choice of ethanol:distilled water to obtain the kneaded products was based on ensuring an optimal complexation efficiency of CDs. One of the methods applied to enhance the complexation efficiency is the use of cosolvents, ethanol being one of them [51,52,53,54]. Ethanol was used as cosolvent, because it can enhance the apparent OLM solubility [32]. Additionally, the presence of ethanol as cosolvent hinders the aggregation tendency of CDs that can affect their binding affinity [53,54]. 

Physical mixtures of OLM and every CD were prepared in the same molar ratio as the KP by mixing the substances in an agate mortar and pestle for 10 min, in a solvent-free manner (PMG) and also by gentle mixing using a spatula (PM). The physical mixtures were passed through a 75 µm size sieve.

### 3.7. FTIR Spectroscopy

UATR-FTIR spectroscopic analysis was carried out using a Shimadzu IRTracer-100 FT-IR spectrometer with ATR device. The data were collected directly from solid samples in the spectral range 4000–400 cm^−1^. Spectra were built up after a number of 16 co-added acquisitions, with a spectral resolution of 2 cm^−1^.

### 3.8. Thermal Analysis

The thermal behavior of OLM, CDs, physical mixtures, and kneaded products was investigated using a Setline TGA instrument (SETARAM, Caluire, France) instrument. Samples with masses of 3–4 mg were placed in aluminum crucibles and analyzed in nitrogen and air atmosphere at a flow rate of 100 mL min^−1^, over the temperature range of 40–500 °C, with a heating rate of 10 °C min^−1^.

### 3.9. Powder X-ray Diffraction

The PXRD pattern of OLM, RM-β-CD, TM-β-CD and their KP and PMG was recorded using a Bruker D8 Advance powder X-ray diffractometer. The XRD patterns were collected at ambient temperature, using CuKα radiation (40 kV, 40 mA) and a Ni filter, over an interval of 10–45° angular domain (2*θ*).

### 3.10. Solubility Profile of OLM/CD Kneaded Products 

The saturation shake-flask method was used to evaluate the changes in OLM solubility as a result of complexation with CDs. For this purpose, an excessive amount of OLM and OLM/CD PMGs and KPs was added in 5 mL of 0.1 M phosphate buffer (pH 7.4) in order to obtain saturated solutions. After shaking for 24 h at room temperature, the solutions were filtered using a 0.45 µm nylon disk filter and appropriately diluted for UV spectrophotometric measurements at 257 nm. The quantification of OLM was performed using the calibration curve. To this end a set of OLM solutions was prepared in 0.1 M phosphate buffer in the concentration range of 2.7–48.6 µg mL^−1^ and scanned with Jena Analytik Specord 250 Plus double beam spectrophotometer in the spectral range of 200-310 nm for obtaining the maximum absorption wavelength. The absorbance of all solutions was recorded at the maximum absorption wavelength, 257 nm. The equation of the calibration curve obtained by plotting the absorbance (*A*) of the solutions versus their concentrations (*C*) is: *A* = 0.04694∙*C* (µg mL^−1^) + 0.00343 (*R* = 0.99992).

## 4. Conclusions

This study is dedicated to investigating the molecular encapsulation of OLM by two methylated CDs. The host–guest interaction of the drug substance with RM-β-CD and TM-β-CD was assessed in solid state and in solution by means of thermal methods, FTIR spectroscopy, UV spectrophotometry, PXRD and solubility studies and also using molecular docking studies. The results of the FTIR studies reveal the disappearance and shift to different wavenumbers of several OLM IR spectral characteristic bands pointing out the existence of an interaction between the drug substance and the two CDs in both PMG and KP and also suggest a possible interaction in PM. The thermoanalytical methods indicate a different thermal behavior of the binary compounds as compared to the parent substance, supporting the findings of FTIR analysis and also emphasize a weaker interaction in PMG and PM products. Furthermore, the binary systems of OLM with the methylated CDs reveal an important reduction in OLM crystallinity, higher for the kneaded products. These different physicochemical properties of the binary products strongly support the hypothesis of the interaction between the drug substance and CDs due to the formation of inclusion complexes in a 1:1 molar ratio, as the phase solubility studies and Job’s method indicated. Solubility analysis highlighted a higher solubilizing effect for RM-β-CD supporting the outcomes of the phase solubility studies, EE and LE analysis, that also indicated a better interaction of OLM with RM-β-CD, thus recommending the OLM/RM-β-CD KP for further research in order to obtain new pharmaceutical formulations with an improved biopharmaceutical profile.

## Data Availability

Data are contained in the manuscript and Supplementary Materials.

## Supplementary Materials

The following supporting information can be downloaded at: https://www.mdpi.com/article/10.3390/molecules29102209/s1.
