# Supplementary material for: Host–Guest Interaction Study of Olmesartan Medoxomil with β-Cyclodextrin Derivatives
Source: Molecules. 2024 May 8;29(10):2209. doi: 10.3390/molecules29102209 (PMC11123892; doi:10.3390/molecules29102209)
Supplement: Supplementary file 1 [file molecules-29-02209-s001.zip › molecules-2980428-supplementary.pdf]

Host–Guest Interaction Study of Olmesartan Medoxomil with β-Cyclodextrin Derivatives

Minodora Andor [1], Claudia Temereancă [2], Laura Sbârcea [3,4], Adriana Ledeți [3,4,*], Dana Emilia Man [1], Cristian Mornoș [1], Amalia Ridichie [3,4], Denisa Cîrcioban [3,4], Gabriela Vlase [5], Paul Barvinschi [6], Angela Caunii [3,4], Renata-Maria Văruț [7], Cristina Maria Trandafirescu [3], Valentina Buda [3], Ionuț Ledeți [2,3,4] and Matilda Rădulescu [1]

1. Faculty of Medicine, "Victor Babeş" University of Medicine and Pharmacy, 2 Eftimie Murgu Square, 300041 Timisoara, Romania; andor.minodora@umft.ro (M.A.); man.dana@umft.ro (D.E.M.); cmornos@cardiologie.ro (C.M.); radulescu.matilda@umft.ro (M.R.)
2. Faculty of Industrial Chemistry and Environmental Engineering, University Politehnica Timisoara, 2 Victoriei Square, 300006 Timisoara, Romania; claudia.temereanca@student.upt.ro (C.T.); ionut.ledeti@umft.ro (I.L.)
3. Faculty of Pharmacy, "Victor Babeş" University of Medicine and Pharmacy, 2 Eftimie Murgu Square, 300041 Timisoara, Romania; sbarcea.laura@umft.ro (L.S.); amalia.ridichie@umft.ro (A.R.); circioban.denisa@umft.ro (D.C.); caunii.angelica@umft.ro (A.C.); trandafirescu.cristina@umft.ro (C.M.T.); buda.valentina@umft.ro (V.B.)
4. Advanced Instrumental Screening Center, Faculty of Pharmacy, "Victor Babes" University of Medicine and Pharmacy Timisoara, 2 Eftimie Murgu Square, 300041 Timişoara, Romania
5. Research Centre for Thermal Analysis in Environmental Problems, West University of Timisoara, Pestalozzi Street 16, 300115 Timisoara, Romania; gabriela.vlase@e-uvt.ro
6. Faculty of Physics, West University of Timisoara, 4 Vasile Parvan Blvd, 300223 Timisoara, Romania; pc_barvi@yahoo.fr
7. Faculty of Pharmacy, University of Medicine and Pharmacy Craiova, 2–4 Petru Rares Str., 200349 Craiova, Romania; rennata_maria@yahoo.com

* Correspondence: afulias@umft.ro

Supplementary Materials

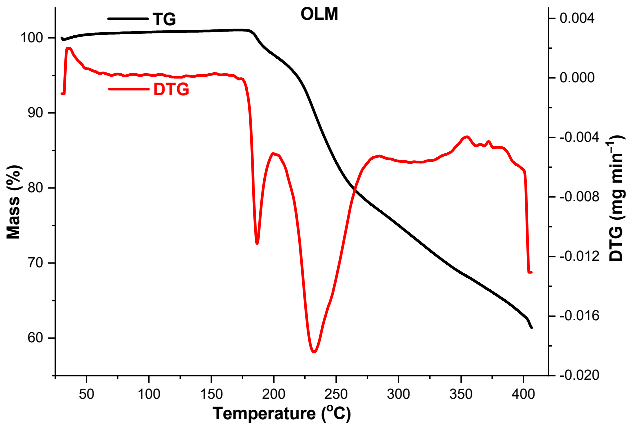

(a)

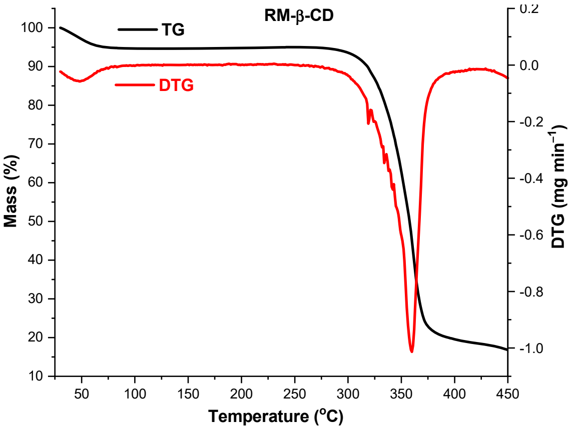

(b)

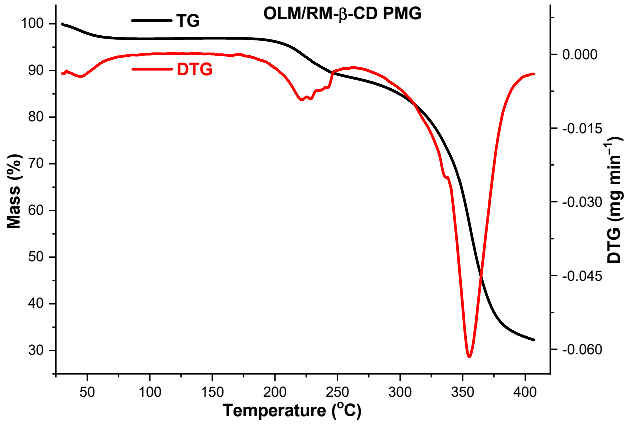

(c)

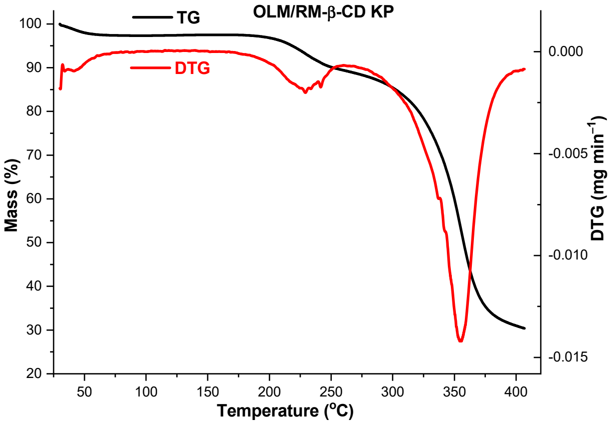

(d)

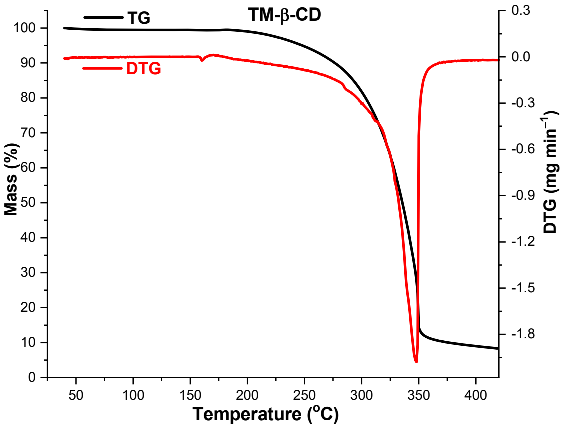

(**e**)

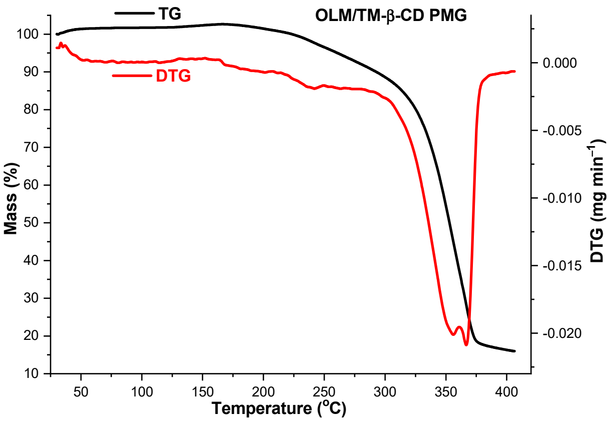

(**f**)

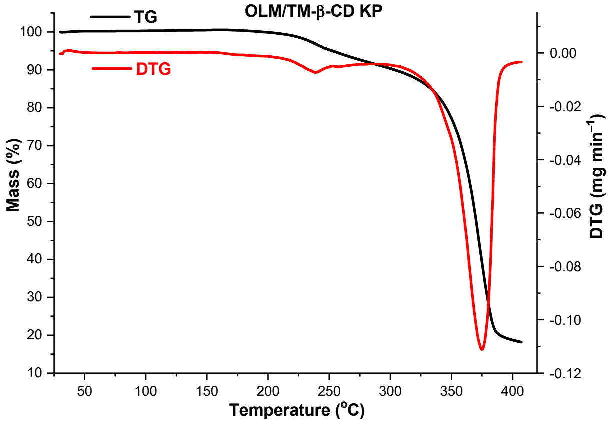

(**g**)

Figure S1. TG/DTG thermal curves of OLM (**a**); RM-β-CD (**b**); OLM/RM-β-CD PMG (**c**); OLM/RM-β-CD KP (**d**); TM-β-CD (**e**); OLM/TM-β-CD PMG (**f**) and OLM/TM-β-CD KP (**g**) in air atmosphere.
